# Effect of the Geometric Uncertainty of Jaw Positioning on the Use of Volumetric Modulated Arc Therapy in Stereotactic Radiosurgery

**DOI:** 10.7759/cureus.87039

**Published:** 2025-06-30

**Authors:** Norifumi Mizuno, Mariko Umeda, Yuito Kato, Takafumi Yamano, Toyokazu Hayakawa, Nobuko Utsumi, Mio Saito, Takeo Takahashi

**Affiliations:** 1 Department of Radiation Oncology, Saitama Medical Center, Saitama Medical University, Kawagoe, JPN

**Keywords:** jaw tracking, metastatic brain tumor, position accuracy, quality assurance, radiosurgery, stereotactic radiotherapy, volumetric modulated arc therapy

## Abstract

Background and purpose

Volumetric modulated arc therapy (VMAT) with a C-arm linear accelerator has been adopted for stereotactic radiosurgery (SRS) for treating brain tumors. Some treatment planning systems (TPSs) generate sequences that align the jaw with the edge of the multileaf collimator (MLC) radiation field during VMAT. However, the jaw exhibits greater geometric positioning uncertainty than the MLC. In this study, we investigated the effect of jaw positioning uncertainty on dose distribution in VMAT-SRS and evaluated the effectiveness of our proposed method.

Methods

The RayStation software (RaySearch Laboratories, Stockholm, Sweden) was used for the TPS and a TrueBeam STx linear accelerator (Varian Medical Systems, Palo Alto, USA) for the C-arm linear accelerator. A target simulating a brain tumor was placed in the phantom, for which three treatment plans implementing VMAT-SRS were created: one using the jaw tracking technique (JT plan), another with a fixed jaw (FJ plan), and a third with the jaw fixed 1 mm outward from the edge of the MLC (FJ1mm plan). The change in the dose-volume parameters relative to the original plan was evaluated when the jaw position at each control point in each plan was systematically changed by ±1 mm.

Results

The maximum changes in the absolute dose received by 99% of the gross tumor volume from the original plans were −7.0%, −5.6%, and −1.1% in the JT, FJ, and FJ1mm plans, respectively. The maximum changes in the absolute dose received by 99% of the planning target volume were −13.1%, −12.0%, and −2.2%, respectively. The ranges of change in the absolute volume of the normal brain receiving a dose greater than 12 Gy for the original plans were −1.3 to 0.6 cm^3^, −0.7 to 0.4 cm^3^, and −0.4 to 0.1 cm^3^, respectively.

Conclusion

Even when the quality control levels recommended by the jaw positioning guidelines were met, target dose variations of >10% were observed depending on the existing VMAT-SRS. Our proposed method was the most robust, with a target dose variation of <3%.

## Introduction

Stereotactic radiosurgery (SRS) is a treatment modality that delivers highly precise, concentrated radiation to localized lesions, thereby minimizing side effects on the surrounding area of the lesion and accurately irradiating the lesion with a large, highly therapeutic dose [1]. SRS is used to treat cerebrovascular disorders, such as cerebral arteriovenous malformations, primary brain tumors (e.g., meningiomas and acoustic neuromas), metastatic brain tumors, and functional disorders (e.g., trigeminal neuralgia). In SRS, the Gamma Knife, a dedicated device that uses a ^60^Co radiation source arranged in a 4π space, has been used for a long time. Later, the CyberKnife (CK), a robot arm equipped with a small linear accelerator, was introduced. More recently, advancements in mechanical technology have led to the development of general-purpose C-arm linear accelerators [2-7]. Dynamic conformal arc therapy and fixed-angle intensity-modulated radiotherapy (IMRT) have been the mainstays for SRS using a linear accelerator [8]. However, volumetric modulated arc therapy (VMAT), a rotating-type IMRT, has gained prominence for its ability to create comparable or superior dose distributions while significantly reducing treatment time compared with CK, DCAT, and IMRT [9-12].

VMAT can modulate the fluence for optimal dose distribution by changing the shape of the irradiation field using a multi-leaf collimator (MLC) for each control point (CP) of the linear accelerator control, such as gantry rotation. Furthermore, a mechanism called “jaw,” which shapes the radiation beam into a rectangle, is placed in the beam path. The jaw compensates for the edges of the complex irradiation field formed using MLC and reduces the dose of radiation transmitted outside the irradiation field. The method of controlling the jaw position for each CP varies across radiation treatment planning systems (TPSs). Some TPSs can create plans where the jaw position aligns with the edge of the irradiation field using MLC. Furthermore, while early VMAT techniques had fixed the jaw position at the maximum aperture position of the MLC, newer jaw-tracking technology adjusts the jaw to dynamically follow the edge of the MLC, reducing the amount of leakage [13,14].

In IMRT using VMAT and other MLC-based fluence modulations, the driving position accuracy of the MLC forming the irradiation field aperture is critical [15-18]. For example, many reports have proposed recommended tolerances for the positional accuracy of MLCs, such as the requirement for 0.2 mm positional and aperture reproducibility [15,16,19,20]. However, few reports have investigated the level of accuracy required for the geometric positioning of the jaws during VMAT, with most studies only conducting comparisons of dose distributions and calculation accuracy across various jaw-tracking planning technologies [13,14]. To the best of our knowledge, no studies have directly investigated the effect of jaw positioning errors on SRS dose distribution. The American Association of Physicists in Medicine (AAPM) Task Group Report on linear accelerator quality assurance categorized the difference between the displayed value and actual jaw position at the same level for conventional irradiation technology and IMRT/VMAT, recommending a difference of ±2 mm or ±1 mm per jaw [17,18]. However, these thresholds may hold too much uncertainty, affecting the positional accuracy of the factors that form the radiation irradiation field, just like the MLC. Even if the recommended level in the guidelines is achieved, the accuracy of the jaw position in VMAT is actually insufficient, the target local control may decrease, or normal tissue toxicity may increase. Furthermore, the impact of the geometric uncertainty of the jaw position during VMAT on the administered dose may be more significant for plans with small irradiation fields, such as SRS. However, there have been no reports directly quantifying dose changes due to geometric positional errors of the jaw in VMAT-SRS, so the impact of jaw positional uncertainty remains unclear.

The objectives of this study were (1) to quantitatively assess the impact of jaw positioning uncertainty on dose distribution in VMAT-SRS and (2) to propose and evaluate a planning method that mitigates these uncertainties. In this study, we investigate the effect of jaw position uncertainty on SRS dose distribution by directly inducing errors for the first time, and further investigate our proposed method. This enables clinicians to ensure treatment quality when using jaws in VMAT-SRS, all to the benefit of patients.

Some of the findings reported here were presented at the 15th Annual Meeting of the Japan Radiosurgical Society in Osaka-shi, Osaka-fu, Japan, held on January 27, 2024.

## Materials and methods

Target design

**Figure 1:**
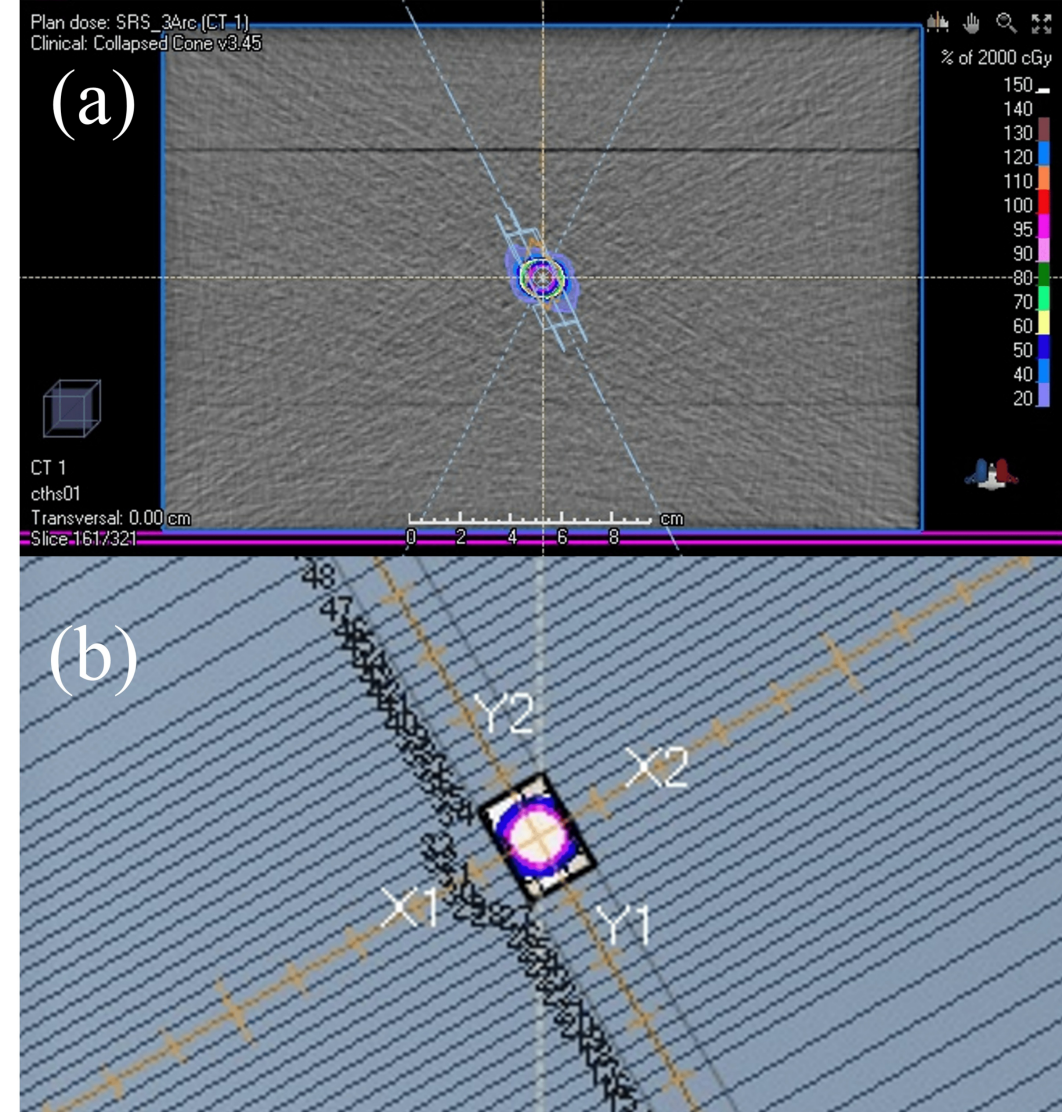
Example of brain stereotactic radiosurgery planning using volumetric modulated arc therapy for a virtual target (a) The target is set at the center of the slab-type phantom. (b) The beam’s eye view shows the target and irradiation field shape from the source side of the linear accelerator. The gross tumor volume is indicated by the pink contour, while the planning target volume is indicated by the blue contour.

To evaluate the effect of jaw geometric positioning uncertainty on dose distribution, a virtual target was created in the treatment planning system to simulate brain SRS, and treatment plans using VMAT were created. The VMAT-SRS plans were generated using the RayStation software (version 6.3.0; RaySearch Laboratories, Stockholm, Sweden). A rectangular phantom with the density of water (30 × 30 × 20 cm) was created on the treatment couch (Figure 1a). Spherical gross tumor volumes with diameters of 1 cm (GTV1) and 2 cm (GTV2) were created in the phantom center as the targets for treatment. Furthermore, the respective planning target volumes (PTV) were generated by expanding each GTV isotropically by 1 mm (PTV1 and PTV2, respectively). The phantom region outside each PTV simulated the normal brain.

Treatment equipment and prescribed dose

We used the TrueBeam STx linear accelerator (Varian Medical Systems, Palo Alto, USA) equipped with an HD 120 MLC (0.25 cm minimum leaf width at the isocenter) as a C-arm linear accelerator model. All treatment plans were composed of 6-MV flattening filter-free photon beams. The isocenter, which is the rotation center of VMAT, was set to the phantom center (i.e., the center of the target; Figure 1b). A total dose of 20 Gy in single fractions was applied as the prescribed dose. The prescription isodose was set to be approximately 70% of the maximum dose. Optimization was performed to minimize the absolute volume of the normal brain receiving a dose greater than 12 Gy (*V*_12 Gy_) [21]. The monitor units were normalized so that the absolute dose received by 99% of the PTV volume (*D*_99%_) would be 20 Gy. The grid size for the dose calculation was set to a constant value of 1 mm, and the collapsed cone convolution superposition was executed as the dose calculation algorithm.

Treatment planning

**Figure 2:**
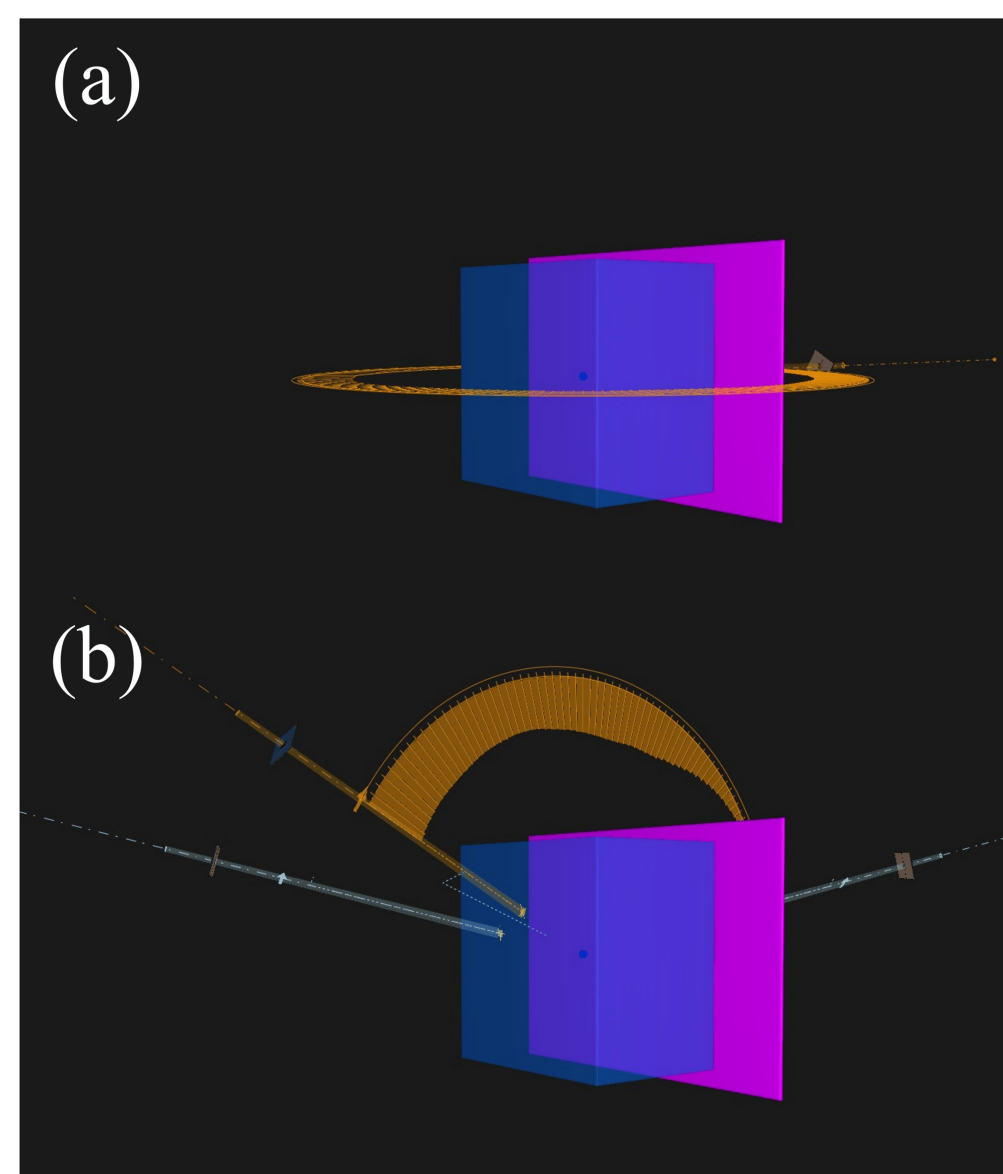
Beam design for stereotactic radiosurgery planning using volumetric modulated arc therapy (a) A coplanar, single full arc of 360° was created with 180 control points (CPs); (b) three couch angles (30°, 90°, and 330°) were applied, and a plan was created for three non-coplanar beams in a 120° arc angle range (60 CPs).

**Figure 3:**
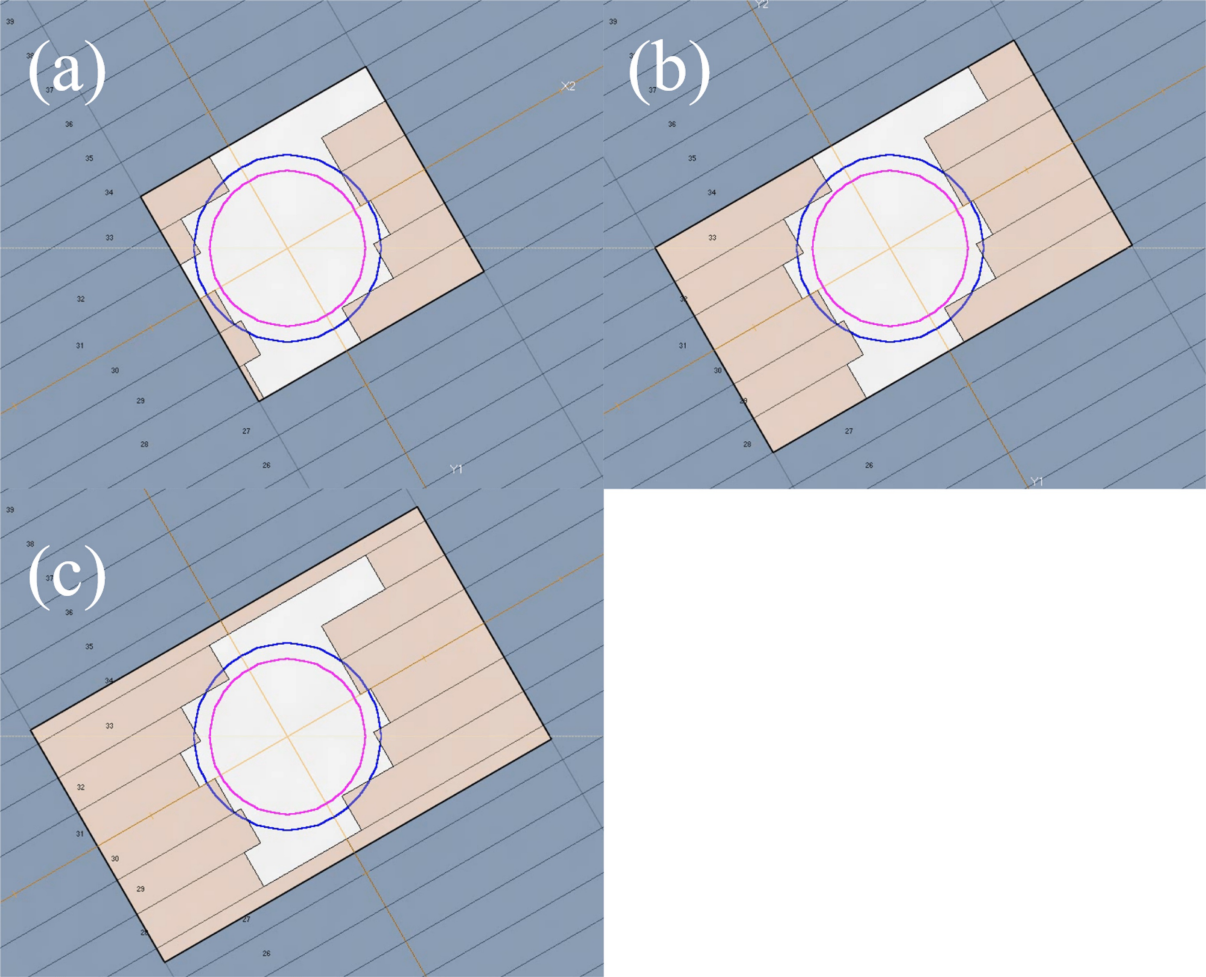
Beam’s eye view showing the difference in the jaw position during volumetric modulated arc therapy (VMAT) beam-on (a) Plan with the jaw-tracking technique turned on (JT plan). (b) Plan with the fixed-jaw technique, in which the jaw does not move dynamically during VMAT (FJ plan). (c) Plan that uses a fixed jaw but allows a margin by moving the jaw 1 mm away from the edge of the irradiation field of the multi-leaf collimator (MLC; FJ1mm plan). The jaw position is indicated by the black rectangular line, while the MLC position is indicated by the brown fill. The pink contour indicates the gross tumor volume, and the blue contour indicates the planning target volume.

To evaluate the effect on the dose distribution of the geometric uncertainty of the jaw position caused by jaw sequence differences during VMAT-SRS, plans using three different control methods were created: (a) one using the jaw-tracking technique (JT plan); (b) another using the fixed-jaw technique, in which the jaw does not move dynamically during VMAT (FJ plan) and involves jaw sequences usually created using the RayStation TPS to strictly follow the edge of the MLC irradiation field and (c) our proposed plan that uses a fixed jaw but allows a 1-mm margin that enables the jaw to move away from the edge of the MLC irradiation field (FJ1mm plan). The third plan is equivalent to adding a margin to the aperture shaped by the MLC that corresponds to the criteria for jaw position accuracy proposed in the guidelines. Figure 2 illustrates the representative beam’s eye view showing the characteristics of the sequence of each plan. VMAT-SRS plans for PTV1 and PTV2 were created for each jaw strategy. The arc rotation range was set to the same value for each plan, and a coplanar 360° single full-arc with 180 CPs was created (Figure 3a). Furthermore, to evaluate the effect of the beam angle, three couch angles (30°, 90°, and 330°) were applied, and a plan for three non-coplanar beams in a 120° arc angle range (60 CPs) was created for PTV1 (Figure 3b). The rotation angle of the collimator was set to 30° for all beams in all plans.

Scenario for the geometric position error of the jaw and dose comparison with the original plan

We created the original VMAT-SRS treatment plan and then uniformly shifted each jaw position (X1, X2, Y1, and Y2) of each CP in the expansion or reduction direction by 1 mm. Both expansion and reduction were applied to all original plans, and the dose distribution was recalculated. Therefore, we simulated the worst-case scenario for jaw position errors under normal quality control conditions while still adhering to the guidelines for linear accelerators. We manually adjusted the settings at all 180 CPs in the original plan on the RayStation TPS to introduce jaw position shifts. In the JT plan, the jaw position changed for each CP of each beam, so it was necessary to shift the values manually one by one for each CP. In the FJ plan and FJ1mm plan, the jaw position was fixed, so the values were changed for each beam. The doses were compared between the plan that caused the jaw position error and the original plan by calculating the relative difference between the near minimum (*D*_99%_) and maximum (*D*_1%_) doses for GTV and PTV. The change in *V*_12 Gy_ was evaluated as a parameter associated with toxicity in the normal brain, and the results were described descriptively.

## Results

Table 1 shows the dose-volume data for the simulated target and normal brain for the jaw position errors in the VMAT-SRS plans. For PTV1 with coplanar beams, the largest differences in the target dose occurred in the JT plan with a jaw position error of −1 mm, with reductions of −7% in GTV *D*_99%_ and −13.1% in PTV *D*_99%_. The next largest dose differences were observed in the FJ plan, with reductions of −5.6% in GTV *D*_99%_ and −12.0% in PTV *D*_99%_. The FJ1mm plan showed the smallest dose differences with −1.1% for GTV *D*_99%_ and −2.2% for PTV *D*_99%_. When coplanar beams were applied to PTV1, no dose differences exceeding 3% from the original plan were observed in any of the plans with a jaw position error of +1 mm. Furthermore, differences in *V*_12 Gy_ of the normal brain remained under 0.5 cm^3^ between each original plan and between plans with jaw position errors. The maximum *D*_1%_ for GTV and PTV was approximately 1% in all scenarios.

**Table 1:** Dose-volume data for the simulated target and normal brain when jaw position errors are introduced in each VMAT-SRS plan with different jaw position controls *Difference > 3%; ** difference > 10% SRS, stereotactic radiosurgery; VMAT, volumetric modulated arc therapy; JT, jaw-tracking technique; FJ, fixed-jaw technique; FJ1mm, fixed jaw with 1 mm margin from the edge of the multi-leaf collimator; GTV, gross tumor volume; PTV, planning target volume; *D*_*x*%_, absorbed dose received by *x*% of the volume; *V*_*x* Gy_, absorbed volume receiving dose greater than *x* Gy

Target	Beam design	Plan	Jaw position error (mm)	Dose-volume data									
				GTV				PTV				Normal brain	
				*D*_99%_ (Gy)	Difference (%)	*D*_1%_ (Gy)	Difference (%)	*D*_99%_ (Gy)	Difference (%)	*D*_1%_ (Gy)	Difference (%)	*V*_12 Gy_ (cm^3^)	Difference (cm^3^)
PTV1	Coplanar beams	JT	None	2310	-	2728	-	2000	-	2725	-	2.6	-
			1	2339	1.3	2737	0.3	2049	2.5	2734	0.3	2.8	0.2
			−1	2148	−7.0*	2700	−1.0	1738	−13.1**	2698	−1.0	2.1	−0.5
		FJ	None	2304	-	2716	-	2000	-	2713	-	2.6	-
			1	2329	1.1	2724	0.3	2044	2.2	2721	0.3	2.8	0.2
			−1	2174	−5.6*	2697	−0.7	1761	−12.0**	2694	−0.7	2.3	−0.3
		FJ1mm	None	2278	-	2665	-	2000	-	2662	-	2.7	-
			1	2284	0.3	2670	0.2	2012	0.6	2667	0.2	2.7	0.0
			−1	2254	−1.1	2657	−0.3	1957	−2.2	2654	−0.3	2.5	−0.2
	Non-coplanar beams	JT	None	2329	-	2800	-	2000	-	2792	-	2.1	-
			1	2361	1.4	2809	0.3	2049	2.5	2802	0.4	2.4	0.3
			−1	2219	−4.7*	2771	−1.0	1839	−8.1*	2760	−1.1	1.7	−0.4
		FJ	None	2320	-	2778	-	2000	-	2771	-	2.2	-
			1	2342	0.9	2784	0.2	2034	1.7	2777	0.2	2.4	0.2
			−1	2262	−2.5	2764	−0.5	1894	−5.3*	2757	−0.5	1.9	−0.3
		FJ1mm	None	2303	-	2738	-	2000	-	2731	-	2.3	-
			1	2310	0.3	2742	0.1	2010	0.5	2736	0.2	2.4	0.1
			−1	2281	−1.0	2732	−0.2	1967	−1.7	2725	−0.2	2.1	−0.2
PTV2	Coplanar beams	JT	None	2210	-	2732	-	2000	-	2724	-	9.8	-
			1	2224	0.6	2739	0.3	2028	1.4	2731	0.3	10.4	0.6
			−1	2128	−3.7*	2718	−0.5	1865	−6.8*	2711	−0.5	8.5	−1.3
		FJ	None	2199	-	2708	-	2000	-	2700	-	10.1	-
			1	2206	0.3	2713	0.2	2015	0.8	2704	0.1	10.5	0.4
			−1	2166	−1.5	2700	−0.3	1916	−4.2*	2691	−0.3	9.4	−0.7
		FJ1mm	None	2190	-	2693	-	2000	-	2685	-	10.3	-
			1	2194	0.2	2697	0.1	2006	0.3	2689	0.1	10.4	0.1
			−1	2183	−0.3	2688	−0.2	1986	−0.7	2680	−0.2	9.9	−0.4

Even when non-coplanar beams were applied to PTV1, the maximum difference in the target dose was observed in the JT plan with a jaw position error of −1 mm. This value was smaller than that for the coplanar beams, with −4.7% for GTV *D*_99%_ and −8.1% for PTV *D*_99%_. Similarly, the evaluated GTV *D*_99%_ and PTV *D*_99%_ were −2.5% and −5.3% in the FJ plan and −1.0% and −1.7% in the FJ1mm plan, respectively. The tendency of normal brain *V*_12 Gy_ in non-coplanar beam plans was similar to that in coplanar beams, with no differences exceeding 0.5 cm^3^ between the plans.

Compared with the above-mentioned results, irradiation of the PTV2 target with coplanar beams resulted in a more significant overall reduction in dose variation within the target. With a −1 mm error in the jaw position, the *D*_99%_ of the GTV and PTV were −3.7% and −6.8% in the JT plan, −1.5% and −4.2% in the FJ plan and −0.3% and −0.7% in the FJ1mm plan, respectively. In targeting PTV2, the differences in *V*_12 Gy_ in normal brain tissue between the original plans remained under 0.5 cm^3^. However, when an error was introduced into the jaw position, the differences in the volume between the original plans and the JT and FJ plans exceeded 0.5 cm^3^.

## Discussion

In this study, the geometric uncertainty of the jaw position was compared among the JT, FJ, and FJ1mm plans, where the FJ1mm plan allowed a margin to the static fitting to cover the worst-case scenario under normal quality control conditions for the jaw. Jaw position uncertainty significantly affected the JT and FJ plans, resulting in a target dose difference of −5% to −10% or more from the original plan with a −1 mm error in the jaw position. The International Commission on Radiation Units and Measurements recommends a target dose accuracy of 5%, which results in a significant clinical disadvantage [22]. Although the jaw position errors introduced are the worst-case scenario for the level of quality control recommended in the AAPM Task Group Report, a pitfall was discovered: performing the recommended quality control measures of the device may not achieve the clinical objectives [15,16]. The JT plan was more significantly affected by the jaw location uncertainties than the FJ plan. This may be because jaw tracking allows the MLC and jaw positions to coincide in more CPs in the MLC sequence. The difference in dose with the original plan was smaller in the position error in which the jaw opening was wider than that in which the jaw opening was narrower because the aperture shape of the original plan was maintained by the MLC in the positive error, whereas, in the negative position error, the jaw itself directly limited the radiation. For the same reason, the range of variation in the direction of the increased target dose is considered to be small, with a maximum change in the *D*_1%_ of approximately 1% for all scenarios for all plans.

**Figure 4:**
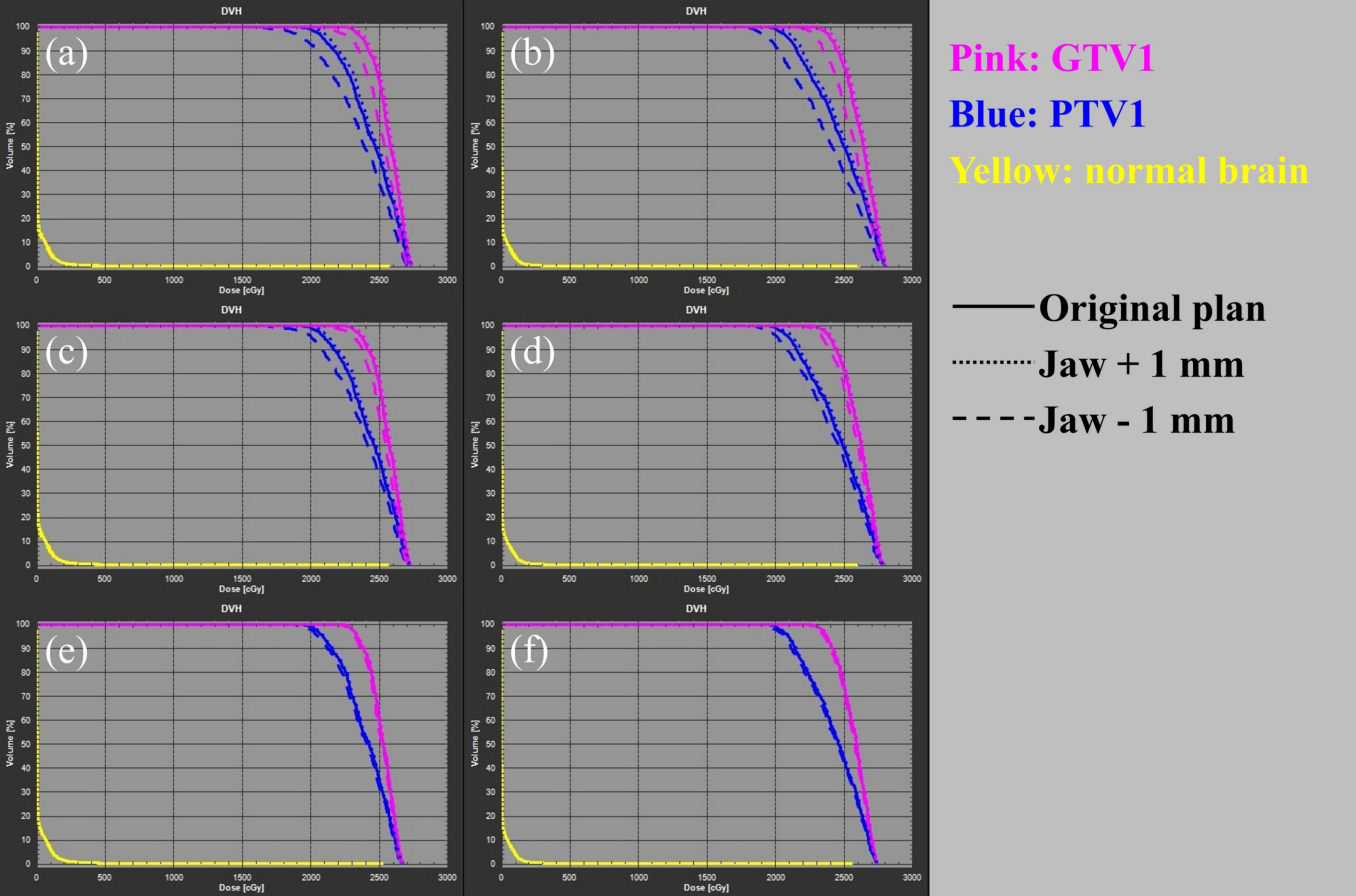
Dose-volume histograms of target and normal organs in stereotactic radiosurgery planning using volumetric modulated arc therapy with jaw position errors (a) Jaw-tracking technique (JT) plan with coplanar beams; (b) JT plan with non-coplanar beams; (c) fixed-jaw technique (FJ) plan with coplanar beams; (d) FJ plan with non-coplanar beams; (e) fixed-jaw with 1 mm margin from the edge of the multi-leaf collimator (FJ1mm) plan with coplanar beams; (f) FJ1mm plan with non-coplanar beams Pink line, histogram of gross tumor volume; blue line, planning target volume; yellow line, normal brain; solid line, original plan; dotted line, plan with a +1 mm jaw position error; dashed line, plan with a −1 mm jaw position error

**Figure 5:**
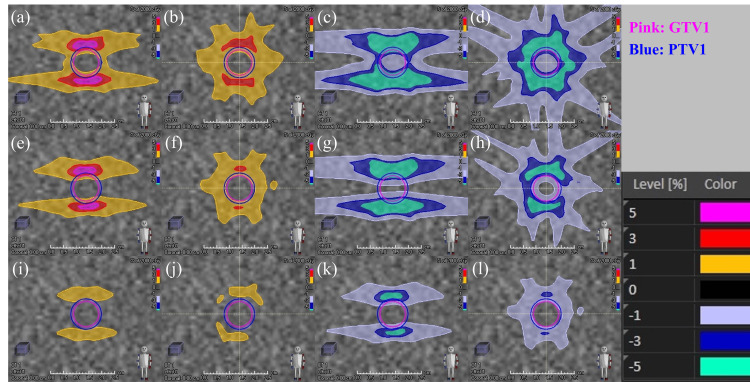
Dose difference from the original plan when uncertainty in the jaw position is introduced in stereotactic radiosurgery planning using volumetric modulated arc therapy (coronal section at the target center) (a) Jaw-tracking technique (JT) plan with coplanar beams, jaw +1 mm; (b) JT plan with non-coplanar beams, jaw +1 mm; (c) JT plan with coplanar beams, jaw −1 mm; (d) JT plan with non-coplanar beams, jaw −1 mm; (e) fixed-jaw technique (FJ) plan with coplanar beams, jaw +1 mm; (f) FJ plan with non-coplanar beams, jaw +1 mm; (g) FJ plan with coplanar beams, jaw −1 mm; (h) FJ plan with non-coplanar beams, jaw −1 mm; (i) fixed-jaw with 1 mm margin from the edge of the multi-leaf collimator (FJ1mm) plan with coplanar beams, jaw +1 mm; (j) FJ1mm plan with non-coplanar beams, jaw +1 mm; (k) FJ1mm plan with coplanar beams, jaw −1 mm; (l) FJ1mm plan with non-coplanar beams, jaw −1 mm The pink contour indicates the gross tumor volume, and the blue one indicates the planning target volume. The dose difference from the original plan is indicated by the color wash.

Our proposed (FJ1mm) plan with a jaw margin reflecting the quality control level of the device proved to be more robust than the conventional jaw position plans for VMAT-SRS usually generated by commercial treatment planning devices (JT and FJ plans). Figure 4 shows the dose-volume histograms of the target and normal organs in VMAT-SRS when jaw position errors were introduced, while Figure 5 shows the dose difference from the original plans in the coronal section at the target center. The histogram of the target doses for the FJ1mm plan (Figures 4e-4f) shows smaller dose variations from the original plan than those for the JT and FJ plans (Figures 4a-4d).

Furthermore, in all scenarios for all plans, the dose difference from the original plan was smaller for the non-coplanar than for the coplanar plan. This may be because, with the use of the collimator angle in this study, the jaw position error accumulated in the head and tail of the target for the coplanar beam (Figures 5a, 5c, 5e, 5g, 5i, 5k), whereas the effect is dispersed for the non-coplanar beam (Figures 5b, 5d, 5f, 5h, 5j, 5l). However, even when non-coplanar beams were applied, a dose difference from the original plan of approximately −5% was observed for GTV *D*_99%_ in the JT plan and −5% to −10% for PTV *D*_99%_ in the JT and FJ plans. In this study, the GTV to PTV margin was set at 1 mm; however, some facilities may set lower margins, raising concerns about the accuracy of GTV doses in the JT and FJ plans [21,23].

Additionally, a wide variety of formulas have been proposed for calculating the GTV to PTV margins; however, they serve different purposes, and some do not account for the mechanical uncertainty of the treatment machine [24,25]. In such cases, a dose reduction of >5% within the GTV to PTV margin observed here may risk reducing the safety margin for other uncertainties that should have been guaranteed. Furthermore, the comparison between PTV2, which has a larger target volume, and PTV1, which has a smaller target volume, indicates that jaw positioning uncertainties should be considered, especially when applying VMAT-SRS to smaller targets. If the JT or FJ plan is applied to smaller targets, the effect of jaw positioning uncertainty may be even larger than that observed in the present study.

Strengths and limitations

To our knowledge, this study is the first to directly simulate the effect of jaw geometric positioning uncertainty on the dose distribution in VMAT-SRS. Furthermore, as far as we know, this report is the first to propose a method (FJ1mm plan) that maintains treatment quality despite jaw positioning uncertainty, the robustness of which was quantitatively demonstrated against conventional methods. Setting margins that uphold jaw quality control standards and positioning them outside the MLC is a practical step that can be implemented at any facility. We believe that our proposed method can effectively ensure the outcome quality of VMAT-SRS plans, which are increasingly being used in clinical practice.

Nonetheless, this study has several limitations. The study was conducted using a limited beam design and single beam energy. Evaluations were performed on only one target of a simple geometrical setup in a phantom. Multiple targets with complex geometries were not explored, and actual patient CT data were not analyzed. Especially for the single-isocenter multi-target VMAT-SRS, the dose difference from the original plan caused by jaw positioning uncertainty may not be as large as differences reported in the present study because of the larger aperture size. When such large apertures are applied, the percentage of the irradiated field that is shielded by the MLC also increases; thus, reducing the MLC transmitted dose with the JT plan may be clinically reasonable, even if some uncertainty in the jaw position is allowed to affect the administered dose. The simulations for jaw positioning uncertainty in this study did not involve the evaluation of the actual linear accelerator; thus, the findings may be excessive for treatment systems in which the jaw position is more strictly controlled. However, the authors believe that this study provides critical preliminary data on the effect of jaw positioning uncertainty in VMAT-SRS.

Although few reports have described planning studies of clinical cases using JT, we believe that our study provides useful information on pitfalls that may occur even when the quality control standards for linear accelerators recommended in the guidelines are met [13-16]. In order to improve the robustness of VMAT-SRS, it may be necessary to review the jaw position management criteria for linear accelerator hardware and propose new quality assurance methods in addition to the TPS-side approach we have proposed.

## Conclusions

This study simulated the effects of jaw positioning uncertainty on VMAT-SRS dose distribution using a treatment planning system. The results demonstrate that even if jaw positioning is quality controlled at the recommended level, significant target dose reduction may occur relative to the planned dose in the worst-case scenario. This clinical inconvenience can be addressed by applying margins to jaw positioning that reflect the institution’s level of quality control to create a robust VMAT-SRS treatment plan, especially for small single targets.
